# Chemotherapy-Related Cognitive Impairment in Patients with Breast Cancer Based on Functional Assessment and NIRS Analysis

**DOI:** 10.3390/jcm11092363

**Published:** 2022-04-23

**Authors:** Noelia Durán-Gómez, Casimiro Fermín López-Jurado, Marta Nadal-Delgado, Demetrio Pérez-Civantos, Jorge Guerrero-Martín, Macarena C. Cáceres

**Affiliations:** 1 Departamento de Enfermería, Facultad de Medicina y Ciencias de la Salud, Universidad de Extremadura, 06006 Badajoz, Spain; casimirolj@unex.es (C.F.L.-J.); jorguerr@unex.es (J.G.-M.); mcaceres@unex.es (M.C.C.); 2 Hospital Universitario de Badajoz, 06006 Badajoz, Spain; mnadal.aoex@yahoo.es; 3 Facultad de Medicina y Ciencias de la Salud, Universidad de Extremadura, Hospital Universitario de Badajoz, 06006 Badajoz, Spain; dperciv@unex.es

**Keywords:** breast neoplasm, dorsolateral prefrontal cortex, near-infrared spectroscopy, cerebral blood flow, cognitive impairment

## Abstract

Background: Chemotherapy-related cognitive impairment (CRCI), or “chemobrain,” isdefined as a phenomenon of cognitive deficits in cancer patients after chemotherapy and is characterized by deficits in areas of cognition, including memory, attention, speed of processing, and executive function, which seriously affect quality of life. The purpose of this study is to investigate the impact of CRCI in breast cancer (BC) patients in chemotherapy treatment (CT+) or not (CT−) and to analyze their relationship with detectable objective changes in cerebral activity during the execution of a phonological and semantic verbal fluency task (PVF and SVF). Methods: An observational, cross-sectional study was carried out at Badajoz University Hospital (Spain). A total of 180 women with BC were included. We used Cognitive Scale (FACT-Cog) for neuropsychological subjective assessment, obtaining scores of perceived cognitive impairment (PCI), and near-infrared spectroscopy system (NIRS) for neuropsychological objective assessment during a verbal fluency task (PVF and SVF), determining alterations in the prefrontal cortex (PFC) assessed as changes in regional saturation index (rSO_2_). Results: A total of 41.7% percent of the patients in the sample had PCI. CT+ was significantly associated with a worse impact in PCI ($\bar{X}$ = 50.60 ± 15.64 vs. $\bar{X}$ = 55.01 ± 12.10; *p* = 0.005). Average rSO_2_ decreased significantly in CT+ ($\bar{X}$ = 63.30 ± 8.02 vs. $\bar{X}$ = 67.98 ± 7.80; *p* < 0.001), and BC patients showed a significant decrease in PVF and SVF on average ($\bar{X}$ = 41.99 ± 9.52 vs. $\bar{X}$ = 47.03 ± 9.31, $\mathrm{and}\;\bar{X}$ = 33.43 ± 11.0 vs. $\bar{X}$ = 36.14 ± 10.68, respectively; *p* < 0.001). Conclusions: Our findings suggest that cognitive impairments in the domain of executive functioning exist among patients with BC who received CT. The results corroborate the hypothesis that CT is an important factor in cognitive impairment in patients with BC, which has been demonstrated by both subjective (PCI) and objective (PVF, SVF, and rSO_2_) neuropsychological measures. The combination of doxorubicin, cyclophosphamide, and docetaxel induce cognitive impairment.

## 1. Introduction

Worldwide, an estimated 19.3 million new cancer cases and almost 10 million cancer deaths occurred in 2020. Female breast cancer (BC) has surpassed lung cancer as the most commonly diagnosed cancer, with an estimated 2.3 million new cases (11.7%) [1]. The prevalence of cognitive impairment (CI) in patients with cancer is currently an important area of research. Chemotherapy-related cognitive impairment (CRCI), or “chemobrain”, is defined as a phenomenon of cognitive deficits in cancer patients after chemotherapy. Increasing evidence exists that chemotherapy treatment (CT) for BC can have acute long-term effects on cognitive functioning [2]. BC patients sustain a number of symptoms (psychoneurological symptoms), and of which, those related to cognitive functioning are increasingly important in clinical practice due to the rise in survival rates and interest in the quality of life of the patient [3]. CRCI is characterized by deficits in cognitive areas, including memory, attention, speed of processing, and executive function, which seriously affect quality of life and work capacity [4,5]. Cross-sectional and longitudinal studies suggest that short-term memory, working memory, and verbal capacity are the most frequently affected, followed by visual-spatial memory, executive functions, and attention span [6]. The severity of reported chemobrain symptoms is variable from subtle to more severe. On occasions, these deficits are subtle in such a way that this subtlety, together with a dependence on tests designed to detect more serious localized deficits, means that cognitive changes are often not detected or are underestimated on a medical level [7].

Many of the studies performed are cross-sectional, and assessment of cognitive functioning is made at a single point in time: during or after CT [8]. Prevalence of CI in cancer patients can be as high as 75% and has been the subject of intense research in recent years [9,10]. Cognitive problems can be detected in up to 30% of patients prior to CT, and up to 75% of patients report some type of CRCI during treatment, which means that cognitive impairment is experienced by a majority of patients with cancer while they are undergoing CT given that they present with some type of neurological complication with regards to cognitive functioning in one or more domains. It has been demonstrated that subjective complaints of CI are most often reported one month after chemotherapy, with some reported perennially although partially ameliorated later on [11].

Several mechanisms have been proposed as being responsible for cognitive changes related to cancer therapy, such as the direct neurotoxic effects of chemotherapeutic agents, indirect inflammatory processes mediated by the immune system, induced hormonal changes, and genetic predisposition [3,12]. Furthermore, symptoms related to cancer, such as fatigue, anxiety, depression, and stress, can have an additional impact on cognitive functioning [13]. In this sense, it cannot be ruled out that among those factors that can increase risk of CI are the direct and indirect effects of CT [14]. Cognitive domains function correctly when brain structure and function are in an optimal condition. Chemotherapy crosses the blood–brain barrier, alters mental functioning, and causes impairment of some cognitive domains [3,14].

When analyzing cognitive function, it is recommended to make a distinction between objective cognitive function, measured by standardized neuropsychological tests, and subjective cognitive function, measured through the number of cognitive problems perceived by patients [15], as we have done in the present work.

We analyzed subjective cognitive function by Functional Assessment of Cancer Therapy-Cognitive Function (FACT-Cog) [16]. Research supports that mild cognitive impairment after chemotherapy is more commonly reported by patients than objectively measured by neuropsychological tests [17] and that FACT-Cog scores are lower during and after CT [18]. This finding is consistent with the diagnosis and the clinical treatment. Furthermore, subjective measurement of cognitive functioning by FACT-Cog and its subscales permits evaluation of experience related with cognitive impairment through examination of the patient’s perspective of their own cognitive functioning, which is something that cannot be detected by means of neuropsychological tests [19]. The majority of the patients undergoing CT report that they suffer a serious diminishing of certain cognitive aspects during and after CT and that, in some cases, these seriously affect their daily life and work. Additionally, the International Cancer and Cognition Task Force (ICCTF) recommends the evaluation of cognitive performance of CT patients [20]. Suggested methods for objective evaluation include verbal fluency tests (VFT) [6,20]. Fluency of word production is dependent not only on verbal functions but also on other cognitive processes, including psychomotor speed, attention, or memory (semantic, episodic, and working memory), as well as the efficiency of executive functions [21].

Objectively, this has also been supported by the results of neuroimaging studies, which suggest cognitive changes especially during and after CT. A variety of self-report (subjective) and objective cognitive assessment tools have been shown to correlate with neuroimaging findings [22]. These show that CRCI is associated with functional changes in the brain, which could be related to diminished cognitive performance [23], with changes in cerebral activation, including the frontal and temporal lobes, in attention span during the execution of active tasks (e.g., multitasking) [24] or tasks that assess verbal fluency [25].

Near-infrared spectroscopy (NIRS), a non-invasive functional neuroimaging technology, widely used in recent years, can measure hemodynamic changes on the surface of the cortices of the bilateral fronto-temporal regions [26,27] by measuring the concentrations of oxygenated hemoglobin (oxy-Hb) and deoxygenated hemoglobin (deoxy-Hb), which are assumed to reflect cerebral blood flow (CBF). There are many types of NIRS devices. The type used in our case provides specifically a measure of rSO_2_ and is highly sensitive to conditions that alter the flow of oxygenated blood to brain tissue. There are indications that NIRS is sensitive enough to also detect small metabolic changes during the performance of cognitive tasks, including VFT by letters or categories [26], and they reveal that VFT was the most widely used task in understanding impaired activation [27] or decreased cognitive performance. Studies suggest that a pattern of structural, perfusion, and functional changes in the brain may be found in BC patients with CT (up to six months) [17].

The main purpose of this study is to investigate the prevalence of CRCI in BC patients during treatment. We hypothesize that, compared to other types of treatment, CT has a greater impact on cognitive performance in patients with BC, and we aim to determine whether the cognitive complaints during treatment are associated with detectable objective changes in cerebral activity by means of NIRS analysis during the execution of a semantic and phonological VFT. To test these hypotheses, we conducted a study of cerebral perfusion in BC patients treated with (CT+) and without (CT−) standard-dose chemotherapy. We predicted that the CT+ group would evidence statistically significant changes in brain perfusion compared to the CT− group during treatment and that these changes would correlate with cognitive performance. Secondarily, we also aim to show the influence of certain chemotherapeutic drugs in this relationship.

## 2. Materials and Methods

### 2.1. Study Population and Setting

We performed an observational, cross-sectional, non-probability study between June 2018 and February 2021 at Badajoz University Hospital (Spain). Women diagnosed with BC in oncological treatment (*n* = 180) were included. BC patients were divided in two groups: CT+ group (*n* = 90) with chemotherapy treatment and CT− group (*n* = 90) without it. All patients were newly diagnosed, had not received any previous oncological treatment, and with a limit since the last CT treatment (CT+ group) no more than two months. All fulfilled the inclusion and exclusion criteria. Inclusion criteria were: (1) not being a minor; (2) being less than 85 years of age; (3) being a patient of Badajoz University Hospital; (4) signing the written informed consent; (5) not having neurological or cognitive impairment that would impede carrying out the assessment; (6) not having previously received treatment for another type of primary cancer; (7) not having a diagnostic record of comorbidity associated with depression, anxiety, and/or cognitive impairment; (8) not having linguistic or communicative barriers; (9) not having a previously diagnosed psychiatric disorder; and (10) not being under psychopharmacological and/or psychotherapeutic treatment.

### 2.2. Procedure

Identification of the cases was carried out at the Medical Oncological Service of the University Hospital of Badajoz. Once patients were identified, the exclusion and inclusion criteria were revised together with the activities programmed for each patient for their recruitment at their next appointment at the hospital. After signing the informed consent, one of our research team conducted a clinical interview. Once the first part of the interview was finished, each participant was given the study questionnaires, and the corresponding measures were taken. All documents and measures were completed face-to-face in the Medical Oncological Service with a previously trained member of the research team. Subsequently, patients’ clinical histories were revised.

### 2.3. Instruments and Measures

#### 2.3.1. Interview and Clinical History

A clinical interview was used to assess self-reported sociodemographic data and clinical and psychological variables of the patients. Patients’ clinical histories were used to assess characteristics of the tumor, pathological anatomy, and therapeutic management variables.

#### 2.3.2. Neuropsychological Assessment

Neuropsychological examinations included both subjective and objective measures chosen according to the Recommendations to Harmonize Studies of Cognitive Function in Patients with Cancer, proposed by the International Cognition and Cancer Task Force [20].

##### Subjective Neuropsychological Assessment: Functional Assessment of Cancer Therapy, Cognitive Scale (FACT-Cog), Version 3

The subjective assessment, consisting of self-report measures of cognitive complaints [28], is regularly used in observational and treatment studies [29,30]. It contains 37 items grouped into four subscales, namely Perceived Cognitive Impairments (PCI), Impact on Quality of Life (QoL), Comments from Others (Oth), and Perceived Cognitive Abilities (PCA), on which mental sharpness, attention and concentration, memory, verbal fluency, functional interference, deficits observed by others, change of previous functioning, and impact of quality of life on the patient were evaluated. Each item was rated based on the experience of the previous week on a scale of five points, from never/not at all (0) to several times a day/a large amount (4). For version 3 of FACT-Cog, the developers of the scale recommend the use of one of the four subscales, the PCI score, as the preferred result [31] and that which is most cited in the literature. Recently, the cut-off points for PCI have been described to classify CRCI: the 18-point PCI (cut-off point < 54) and the complete 20-item PCI (cut-off point < 60) were examined. Both PCI-18 and PCI-20 showed good discriminatory capacity for classification of CRCI [32,33]. In the present study, PCI-18 was used.

We requested permission to apply the questionnaire previously adapted for use with Spanish-speaking cancer patients [16].

##### Neuropsychological Objective Assessment: VFT and Procedure

A VFT was employed to test cognitive functions while assessing prefrontal cortex (PFC) hemodynamics by NIRS. The VFT evaluation was divided into two tests: (1) a verbal semantic fluency test (SVF), in which the subject is asked to name all the elements within a given semantic category (animals, plants, and tools), and (2) a phonological verbal fluency test (PVF), in which the subject is asked to say all the words that begin with a particular syllable or letter (pa, la, ro, o, z). Each block lasts 60 s, using a period of 20 s for each semantic category, syllable, or given letter, with a rest interval of 10 s every time a new one is introduced. Incorrect responses included saying “pass”, listing peoples’ names, repeating words, or producing grammatical variations of a previous word. Behavioral performance was assessed as the total number of correct words generated.

##### Neuropsychological Objective Assessment: NIRS Measurements and Procedure

The INVOS 5100 Cerebral Oximeter (Somanetics Corporation, Troy, MI, USA) was used to measure rSO_2_ in the dorsolateral PFC bilaterally. NIRS provides measures of (oxy-Hb) and (deoxy-Hb). Near-infrared light absorption by (oxy-Hb) and (deoxy-Hb) was calculated using a modified Beer–Lambert Law. The relative amounts of both are used to calculate rSO_2_, and their cortical concentration changes are used as an indirect indicator of regional brain activation. The relationship between a decrease in (deoxy-Hb) (and consequently an increase in rSO_2_) and an increase in the blood-oxygenated-level dependent signal of NIRS is a measure of cerebral activation. rSO_2_ was calculated assuming an arterial to venous blood ratio of 25:75%. The INVOS provides real-time measurement and a display of rSO_2_ in the microvasculature beneath the sensor. The two disposable LED sensors alternated between emitting 710 and 830 nm wavelengths of light that are absorbed by hemoglobin. The two receiving optodes were 3 and 4 cm in distance from the LED. Light traveling from the sensor’s light emitting diode to either a proximal or distal detector permitted separate data processing of shallow and deep optical signals.

Before the beginning of the task, participants were instrumented with sensors for the left and right frontal lobes at the dorsolateral level of the PFC. The sensors were correctly secured in place.

rSO_2_ was measured while the participant performed a VFT under the following conditions: (1) rest (pre-test baseline, 1 min); (2) VSF (2 min); (3) VFF (2 min); and (4) rest (post-task baseline, 1 min). The measurements obtained will be named as follows: CT+ group by rSO_2_-CT+_1_, rSO_2_-CT+_2_, rSO_2_-CT+_3_, rSO_2_-CT+_4_, and average rSO_2_-CT+ and CT− group by rSO_2_-CT-_1_, rSO_2_-CT-_2_, rSO_2_-CT-_3_, rSO2-CT-_4_, and average rSO_2_-CT−. The measurements obtained will be named as average rSO_2_. Throughout this period, the subject sat on a comfortable chair in a room that was illuminated by daylight. The sitting position is necessary to ensure comparability across studies since spontaneous physiological oscillations, which are posture dependent, can influence the NIRS signal quality. A mean was determined from the values recorded from two channels in the dorsolateral area of the PFC.

#### 2.3.3. Statistical Analysis

The variables were studied both from a descriptive and an inference point of view. The socio-demographic and clinical characteristics of the total number of enrolled women were analyzed with descriptive statistics in terms of mean ± standard deviation and percentages. Some results are expressed in terms of median and interquartile range. A multiple correspondence analysis (MCA) was performed to detect the principal associations in the consumption of medication. The inference was carried out by Student’s *t*-test, one-way ANOVA test, and chi-square test as required. The correlation between the quantitative variables was calculated by means of Pearson’s correlation coefficient.

All statistical analyses were performed using IBM Corp. Released 2013. IBM SPSS Statistics for Windows, Version 22.0. Armonk, NY: IBM Corp. For all analyses, the α-level was set at *p* ≤ 0.05.

## 3. Results

A total of 180 women with an average age of 53.87 ± 10.54 years (range 29–83 years), diagnosed with BC under initial treatment, took part in our study. The sociodemographic and clinical variables of the sample are detailed in Table 1.

### 3.1. Comorbidity and Obstetric-Gynecologic Antecedents

The percentage of patients with a concomitant condition in addition to the oncological pathology and accepted according to the inclusion criteria was 66.7% (*n* = 120). With regard to obstetric-gynecologic antecedents, 76.1% (*n* = 137) of the women were menopausal: 52.2% (*n* = 94) had natural menopause, 17.2% (*n* = 31) had drug-induced menopause, and in 6.6% (*n* = 13) menopause had been induced by a previous intervention unrelated to BC.

### 3.2. Location of the Tumor, Anatomopathological Characteristics, and Family Antecedents

Prevalence of the disease was greater in the left breast (54.4%, *n* = 98). With regard to the TNM of the sample, it was found that the most frequent stage was stage II with 33.8%, followed by stage I with 31.6%, 17.8% for stage III, 11.6% for stage IV, and lastly 10% for stage 0. The most frequent histological grades were grade III (56.1%, *n* = 101) and grade II (27.8%, *n* = 50). Regarding the immunohistochemical study, positive estrogen receptors (ER) were present in 80.6% and progesterone receptors in 59.4%. HER2 positives were 36.7%, HER2 negatives 52.8%, and triple negative 10.5%. The Ki67 value had an average of 25.12 ± 18.24.

A total of 71.1% (*n* = 128) of the sample had family antecedents of different types of cancer, of which 34.4% (*n* = 62) were breast cancer.

### 3.3. Therapeutic Management

The stage of illness of the patients in the CT+ group was significantly more advanced than the patients in the CT− group (χ^2^ = 12.656; *p* = 0.010), as was expected given the current protocols of treatment.

In the CT+ group, the average number of cycles in our sample was 6.05 ± 7.45. We were able to establish a cut-off point at ≥ 4 cycles, which gave the following result: 58.9% (*n* = 53) of patients had received fewer than 4 cycles, and 41.1% (*n* = 37) had received 4 or more cycles.

With regard to CT treatment and antineoplastic agents (Table 2), we highlight the combinations with the highest results. For the joint analysis, medication consumed by less than 5% of the patients was not taken into account. MCA was performed to detect the principal associations in the consumption of medication; these associations were subsequently confirmed by the χ^2^ test. We highlight those that were highly significant (*p* < 0.001) (Table 3).

The patients in the CT+ group were, therefore, treated with regimens of standard dose polychemotherapy, and the majority of the patients received a combination of two or three cytotoxic agents, such as doxorubicin (anthracycline agent), cyclophosphamide (alkylating agent), and docetaxel (taxane).

### 3.4. Cognitive Impairment, NIRS Measures, and VFT

Regarding the functional cognition evaluation, 41.7% (*n* = 75) of the sample had PCI (cut-off points < 54). In the CT+ group, 43.1% (*n* = 39) patients suffered PCI, which was 22% of the total sample. In CT−, only seven patients (7.7%) were found to have PCI.

There was no significant relation between the FACT-Cog scales, PCI, OTh, PCA, QoL, and PCI (<54) for any of the socio-demographic variables studied and other clinical variables by *t*-test, one-way ANOVA, or chi-square test as appropriate. The functional cognitive capacity of our patients is independent of age, marital status, educational level, employment situation, and responsibility for caring for the elderly and/or dependents. There were no significant differences in comorbidity, obstetric-gynecologic antecedents, location of tumor, anatomopathological characteristics, and family antecedents.

**Figure 1:**
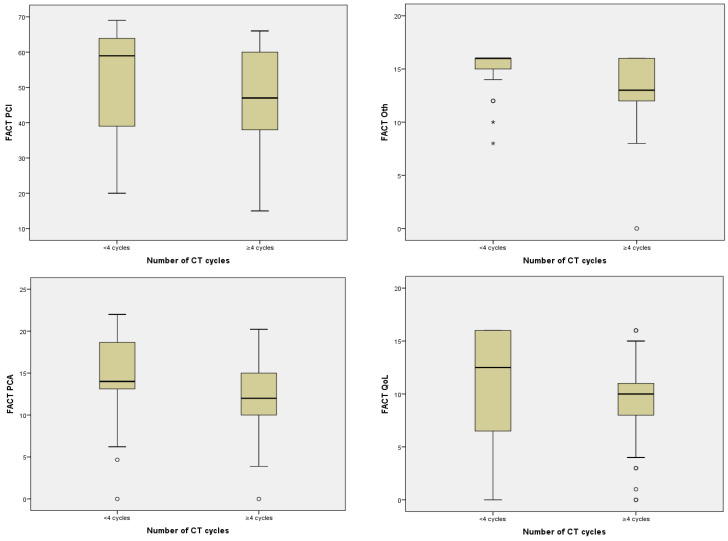
Relationship between number of CT cycles and FACT-Cog scales.

Treatment with CT was significantly associated with worse impact in PCI (*p* = 0.005), Oth (*p* = 0.026), PCA (*p* = 0.039), and QoL (*p* = 0.034) although this was not the case with the rest of the treatments (Table 4). In fact, those patients who received ≥4 cycles of CT correlated inversely with the four scales of cognitive functioning in such a way that the higher the number of cycles received, the worse the scores on the mentioned scales: PCI (*p* = 0.002), Oth (*p* < 0.001), PCA (*p* = 0.001), and QoL (*p* = 0.023) (Table 4, Figure 1). Additionally, taking into account the PCI cut-off point, a statistically significant relationship was found between receiving ≥4 cycles of CT and a clinically significant PCI (*p* = 0.030).

**Figure 2:**
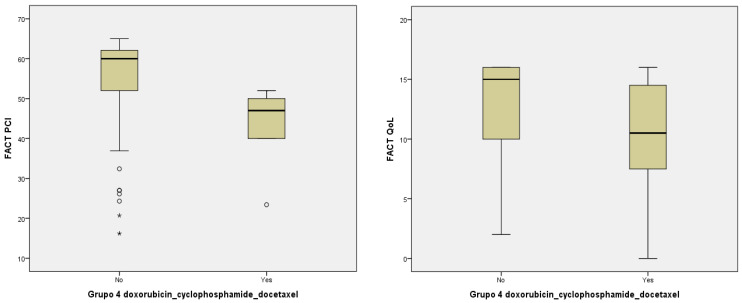
Relationship between PCI, QoL, and pharmacological group 4 (doxorubicin–cyclophosphamide–docetaxel).

With regard to the pharmacological regimen of CT and/or neoplastic agents, there was a statistically significant relationship between pharmacological group 4 and worse impact in PCI and QoL (doxorubicin–cyclophosphamide–docetaxel) (*p* = 0.013, *p* = 0.019) (Table 4) although this was not so with the rest of the pharmacological agents in groups 1, 2, 3, 5, and 6 (Figure 2).

Considering the objective neuropsychological variables, there was no significant relation between PVF, SVF, and rSO_2_ for any of the socio-demographic variables studied, namely comorbidity, obstetric-gynecologic antecedents, location of tumor, anatomopathological characteristics, and family antecedents, and other clinical variables by *t*-test, one-way ANOVA, or chi-square test as appropriate. Instead, we found this significant relationship with variables related to therapeutic management: CT and number of cycles although we also found no relationship with the six most common groups of antineoplastic agents.

**Figure 3:**
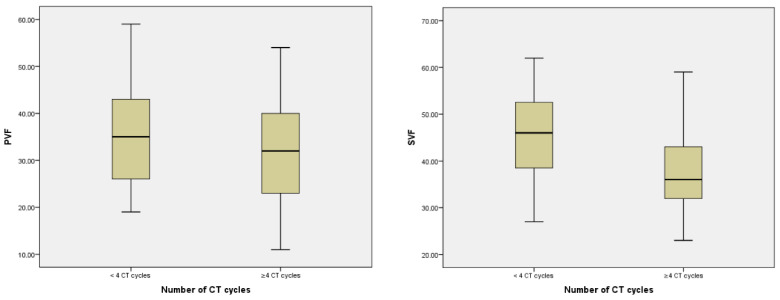
Relationship between number of CT cycles, PVF, and SVF.

CT+ patients showed a significant decrease in PVF and SVF (*p* < 0.001) compared to the CT− group (Table 5). Furthermore, PVF and SVF scores correlated inversely with the number of cycles received (*r* = −0.332, *p* < 0.001; *r* = −0.154, *p* = 0.040, respectively). The group of patients who received > 4 cycles showed a clear worsening both in PVF (*p* < 0.001) and in SVF (*p* = 0.004) (Figure 3).

Something similar occurred with the average of the rSO_2_ (Table 5), which clearly correlated with a significant decrease in PVF (*r* = 0.535, *p* < 0.001) and SVF (*r* = 0.485, *p* < 0.001) in CT+. Additionally, the CT+ group showed a significant decrease in rSO_2_ (*p* < 0.001) with respect to the rest of the treatments.

Evolution of rSO_2_ measures is shown in Table 6. The means on the left, the right, and both sides as well as the correlation between measures are shown on columns of the table. Finally, the last two rows show the means of the three measures in CT+ and CT− groups.

**Figure 4:**
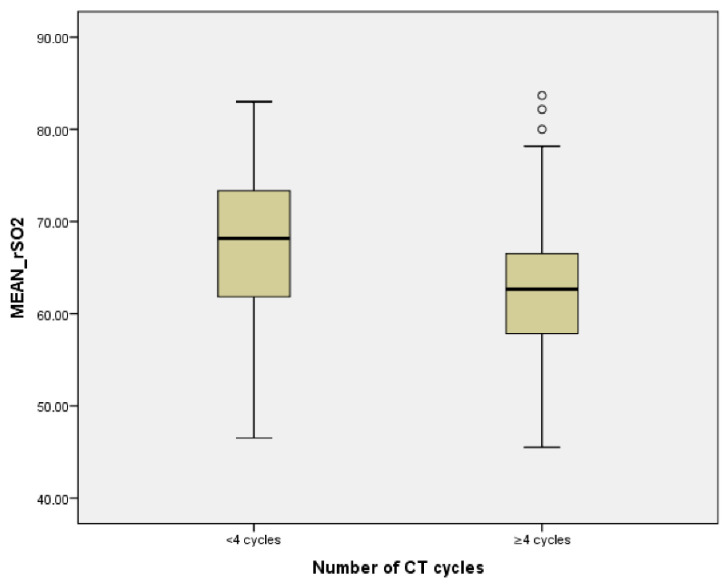
Relationship between number of CT cycles and average rSO_2_.

The average of rSO_2_ correlated inversely with the number of cycles of CT (*r* = −0.225, *p* = 0.002) and with the group of patients with more than four cycles of treatment (*p* < 0.001) (Figure 4).

At the same time, the scales of cognitive functioning PCI, PCA, Oth, and QoL correlated with the PVF scores (*r* = 0.516, *p* < 0.001; *r* = 0.675, *p* < 0.001; *r* = 0.452, *p* < 0.001, respectively), the SVF scores (*r* = 0.630, *p* < 0.001; *r* = 0.648, *p* < 0.001; *r* = 0.153, *p* = 0.041), and with the rSO_2_ average *r* = 0.650, *p* < 0.001; *r* = 0.395, *p* < 0.001; *r* = 0.405, *p* < 0.001, respectively) (Table 7) and also with the clinically significant PCI (<54, *p* < 0.001), thus leading us to establish a direct relationship between the subjective and the objective measures: worse scores in PCI result in a worsening of the rSO_2_ index and a worse performance in the PVF and SVF tests.

## 4. Discussion

To our knowledge, the present study is the first to attempt to evaluate cognitive impairment in the brain functions of BC patients by direct assessments of cerebral hemodynamic reactivity measured by PFC oxygenation (decreased rSO_2_) using a non-invasive NIRS method during treatment. We can state therefore that cognitive complaints in the group of CT+ BC patients could be predictive of cognitive decline given that we found a significant relationship between subjective measures (Fact-Cog PCI, PCA, Oth, and QoL) and objective measures (VFF, VSF, and neuroimaging measures by rSO_2_ index) treated with CT, which is in contrast with previous studies [34], which reported no evidence of this relationship. Therefore, it is important to assess cognitive complaints, including impact on QoL. This could make it possible to detect patients at risk of decline and to anticipate cognitive alterations by proposing adapted interventions, such as cognitive training [34,35].

Approximately 43.1% of the patients in the CT+ group had subjective cognitive decline. A recent meta-analysis suggests that cognitive impairment may impact up to one in three patients at a level that is clinically significant [36]. Some studies report that even if only perceived, PCI significantly alters QoL and should be considered as such when assessing BC patients’ needs [37]. Furthermore, PCI was correlated with worsening in objective neuropsychological test scores (PVF, SVF, and average rSO_2_). The results show that decline in cognitive function not only appeared in the objective neuropsychological test but that the subjective FACT-Cog test also came to the same conclusion. All FACT-Cog scores decreased significantly. Our results on the subjective assessment of cognitive complaints demonstrate that there is a correlation between the group of BC patients undergoing CT who received more than four cycles of CT with the four subscales, in agreement with the studies that examined this relationship. Lange et al. (2016) [34] showed that the CT+ group had a significantly greater increase in subjective cognitive complaints after treatment than the CT− group, and that healthy groups on the PCI subscale and a clinically significant subjective decline in the PCA subscale score was observed mainly in the CT group using FACT-Cog. In the study by Tong et al. (2020) [11], CT+ patients performed significantly worse after chemotherapy on FACT-Cog. In our CT+ group, the patients had an average of treatment of 11.45 ± 8.33 months with an average of CT cycles of 7.02 ± 8.65, which suggests that the existence of PCI is likely given that it has been shown to be one of the most commonly occurring symptoms among women with BC during the first 18 months of therapy [38] and that this symptom is relatively stable throughout the treatment. Some studies have even found that cognitive performance of BC patients significantly decreased one month after CT [11].

We found in our study a conclusive relationship between CRCI and PVF/SVF, which is in agreement with published results. The results of a meta-analysis published by Lindner et al. (2014) show that patients treated with CT suffer a decline in the cognitive functions needed to perform tasks assessing verbal fluency, that is, attention capacity and selectivity, as well as delayed immediate verbal memory compared to healthy individuals [6]. These researchers also noted deficits in executive functions among BC survivors, such as working memory, cognitive flexibility, or multitasking [39]. Specifically, with regard to the relationship between CRCI and VFT in its PVF and SVF dimensions, our results show a clear, direct association between their scores. In this respect, the investigation conducted by Quesnel et al. (2009) [40] shows a decrease in PVF in women with BC immediately after and 3 months after completing adjuvant CT, and Hermelink et al. (2007) [41] reported a diminishing of PVF and SVF before finishing neoadjuvant CT treatment. Similarly, research by Jansen et al. (2011) [14] found that 52% of the women in their study experienced a decrease in a variety of cognitive domains and noted that these alterations in cognitive functioning occurred during active treatment with CT or immediately after completing it. The domains that were most affected were visual-spatial ability, motor function, attention, immediate memory, and language. Freeman and Broshek (2002) [42] also found that the cognitive performance (in language) of the active chemotherapy group was significantly below the post-treatment group and that patients had a significantly lower standard score in VFT. In recent investigations, it has been demonstrated that SVF is significantly lower in women treated with adjuvant CT than in healthy women [43]. Additionally, recent prospective studies [44] that have related CT and BC have shown that although CT groups and healthy controls did not differ in the majority of the neuropsychological tests, they did differ significantly in PVF. Even the results from other studies [45] in which chemotherapy does not decrease verbal fluency demonstrate, as in our case, a negative impact on semantic memory.

These findings support our hypothesis, and we may explain our results on the relation between CRCI and the decrease in SVF/PVF as follows: (1) since the aim of SVF is to verify language, semantic memory, and executive functions, by evaluating recuperation capacity of words established in the long-term memory [11] and (2) that in the PVF, patients must maintain instructions in working memory and suppress semantically related words while adapting novel search strategies (this measures verbal and executive control ability), we can identify CRCI in the CT+ group. This implies that CT affects specific domains of executive functioning [44]. These findings suggest that CT-treated patients are vulnerable to cognitive control and monitoring [46,47], and they deviate somewhat from the findings that cognitive impairments in breast cancer patients occur independently of CT [48,49].

Continuing with this relationship, we have demonstrated objectively that a clear worsening in NIRS scores exists: the CT group showed a significant decrease in average rSO_2_, which also correlates with a worsening in the scores for the PVF and SVF tasks. On this point, we should emphasis that the neuroimaging studies carried out to date were not done with NIRS but rather with functional magnetic resonance (fMRI) in most cases. NIRS assessments have been demonstrated to provide a metric of cognitive activation similar to fMRI during cognitive performance tasks [50]. With regard to our investigation, the results of the neuroimaging study indicate that CT is associated with functional and structural changes in the PFC, which is a crucial neural region for executive functioning [39,51]. It has also been demonstrated that activation of the prefrontal lobe was reduced in women with BC after CT while tasks of executive functioning tests were being carried out [52] and that the density of the gray matter in the left middle and superior frontal gyri in women with breast cancer was lower 1 month post chemotherapy [39,53]. The prospective study performed by McDonalds et al. (2012) revealed decreased activation in inferior frontal regions 1 month post chemotherapy [53]. In our case, the measures obtained by NIRS are based on the following principle: an increase in CBF, an increase in (oxy-Hb), and a decrease in (deoxy-Hb) are all seen in active brain regions while people are participating in cognitive tasks; this principle is the basis of neuroimaging techniques such as NIRS. Although they used another neuroimaging technique (fMRI), Nudelman et al. (2014) [54] provide evidence that CT is associated with alterations in cerebral perfusion independently of the effects of the cancer. Statistically significant hyperperfusion was found in the superior and posterior regions after CT, but this was not observed in patients who had not received CT or in controls. The most relevant results from Bai et al. (2020) [18] suggested that the effect of CT on cerebral structure and function involve the frontal lobe and is accompanied by changes in cerebral activity.

Tao et al. (2016) [55] conducted a study of impairment of the executive function in BC patients receiving CT treatment by fMRI and concluded that that CT treatment may influence functional changes in the prefrontal cortex, resulting in impaired executive function in BC patients. They showed that BC patients had impairments measures in comparison with controls and that abnormal brain functional connectivity was observed in these patients. Moreover, the regions of abnormal brain functional connectivity were focused on the fronto-temporal lobes. This suggests that altered brain function connectivity may be contributing to cognitive deficits in BC patients.

Much evidence exists on the effect of modern chemotherapy regimens on cognition, including cyclophosphamide, anthracyclines, and taxanes, in young BC patients [14,56]. With regard to therapeutic management of the patient, in our study, we found that the combination of doxorubicin, cyclophosphamide, and docetaxel induce cognitive impairment. There are studies that consider this relationship as conclusive [57]: the appearance of significant cognitive decline in patients treated with CT based on doxorubicin, with emphasis on the decrease in executive function, language, short-term verbal memory, and processing speed capacity, establish the possibility that subsets of patients exist that are more or less susceptible to cognitive decline mediated by doxorubicin The results of preclinical studies also provide evidence that the combination of doxorubicin and cyclophosphamide negatively affect hippocampal neurogenesis [58], and they impact synaptic plasticity and cause aging of molecules [59], inducing cognitive impairment. The importance of identifying doxorubicin side effects is that it can guide the development of derivative treatments that minimize side effects while maintaining anti-tumorigenicity [57]. Some preclinical studies, on the other hand, have shown that docetaxel can induce cognitive impairment [60,61].

Our study is not without limitations. We evaluated the acute, short-term effects of CT; we did not include the analysis of long-term cognitive performance of the patient groups. A longitudinal design, in which variables are measured before, during, and after treatment, would have identified with greater precision the factors that can affect CRCI. Every effort was made, however, to include the highest possible number of patients from our hospital during the recruiting period. Another limitation is the absence of a control group. In future studies, the use of a control group will help to further differentiate the improvements in the scores from the tests on the effects of the treatment.

## 5. Conclusions

Although much remains to be done in the objective evaluation of CRCI in the BC population, our study has shown that women undergoing CT treatment do so with objective and subjective cognitive costs. Our findings suggest that cognitive impairments in the domain of executive functioning exist among patients with BC who received CT. The results corroborate our hypothesis that CT is an important factor in cognitive decline in BC patients. We have not only reported subjective cognitive evaluations, but objective cognitive assessments were also performed to explore the actual underlying conditions. We found a direct relationship between subjective and objective measures in CT group: lower scores in PCI determine a worsening of the rSO_2_ index and a worse performance in the PVF and SVF tests. It is also possible that the degree and brain areas of attention, memory, and executive function may be dependent on the duration, varying combination, or total dose of chemotherapy. Consequently, future studies should focus on identifying those groups of patients that are at greater risk of developing CRCI and who are, additionally, in treatment with determined antineoplastic drugs, such as doxorubicin, cyclophosphamide, and docetaxel, with the aim of performing cognitive interventions before and after treatment. With this in mind, reducing the morbidity associated not only with the illness but also with the treatment for the illness should be an area for wider exploration.

On the clinical front, efforts must be made to raise awareness among doctors, patients, and their carers of the risk of chemobrain in order to prompt more efficient vigilance of subtle deficits, which otherwise would go unnoticed. Providing relevant strategies of managements for these negative consequences may help increase the long-term quality of life of patients with BC. Refinements in the sensitivity of diagnostic tools to detect mild cognitive decline and the use of non-invasive neuroimaging techniques such as NIRS would improve chemobrain diagnoses.

## Figures and Tables

**Table 1:** Sociodemographic data and characteristics of the sample.

		N = 90	N = 90
Variable	Categories	CT+ N (%)	CT− N (%)
Marital status	Married	64 (71.7)	66 (73.3)
	Single	9 (10)	12 (13.3)
	Divorced	6 (6.7)	8 (9)
	Widowed	11 (12.2)	4 (4.4)
Education level	No studies	9(10)	5 (5.5)
	Elementary school	30 (33.3)	25 (27.8)
	Middle school	13 (14.4)	11 (12.2)
	High school	15 (16.7)	24 (26.7)
	Higher education	23 (25.6)	25 (27.8)
Employment situation	Currently in employment	8 (8.9)	12 (13.3)
	Temporary sick leave	43 (47.8)	31 (34.4)
	Permanent sick leave	9 (10)	5 (5.6)
	Unemployed	20 (22.2)	25 (27.8)
	Retired	10 (11.1)	17 (18.9)
Tumor staging	0	4 (4.4)	5 (5.6)
	I	18 (20)	39 (43.3)
	II	31 (34.5)	30 (33.3)
	III	24 (26.7)	8 (8.9)
	IV	13 (14.4)	8 (8.9)
Grade	1	18 (20)	25 (27.8)
	2	23 (25.6)	40 (44.4)
	3	49 (54.4)	49 (27.8)
Molecular subtype	Luminal A/Luminal B HER2 negative-like	47 (52.2)	60 (66.7)
	Luminal B HER2 positive-like/HER2-type	33 (36.7)	30 (33.3)
	Triple negative	10 (11.1)	0 (0)3
Menopause	Natural	42 (46.7)	52 (57.8)
	Drug-induced menopause	25 (27.8)	6 (6.7)
	Intervention-induced menopause	3 (3.3)	9 (10)
	Reproductive stage	20 (22.2)	23 (25.5)
Surgical treatment	Conservative surgery	41 (45.6)	76 (84.5)
	Uni- or bilateral mastectomy	23 (25.5)	11 (12.2)
	Without surgical treatment	26 (28.9)	3 (3.3)
Chemotherapy cycles	Chemotherapy cycles < 4	53 (58.9)	
	Chemotherapy cycles ≥ 4	37 (41.1)	

**Table 2:** CT+ treatment: antineoplastic agents.

L ANTINEOPLASTIC AND IMMUNOMODULATING AGENTS		
ATC Classifications	*n*	(%)
L01 ANTINEOPLASTIC AGENTS		
L01A ALKYLATING AGENTS		
L01AA Nitrogen mustard analogues		
01 Cyclophosphamide	53	58.9
L01B ANTIMETABOLITES		
L01BA Folic acid analogues		
01 Methotrexate	1	1.1
L01BC Pyrimidine analogues		
02 Fluorouracil	1	1.1
06 Capecitabine	2	2.2
L01C PLANT ALKALOIDS AND OTHER NATURAL PRODUCTS		
L01CD Taxanes		
01 Paclitaxel	6	6.7
02 Docetaxel	54	60
L01D CYTOTOXIC ANTIBIOTICS AND RELATED SUBSTANCES		
L01DB Anthracyclines and related substances		
01 Doxorubicin	36	40
03 Epirubicin	3	3.3
L01X OTHER ANTINEOPLASTIC AGENTS		
L01XA Platinum compounds		
02 Carboplatin	9	10
L01XC Monoclonal antibodies		
03 Trastuzumab	14	15.6
13 Pertuzumab	6	6.7
14 Trastuzumab emtamsine	3	3.3
L01XX Other antineoplastic agents		
41 Eribulin	2	2.2

**Table 3:** Results of the most frequent antineoplastic agents in the study sample.

Group	Drug	*n*	%
1	Docetaxel	10	11.1
2	Cyclophosphamide + Docetaxel	14	15.5
3	Cyclophosphamide + Doxorubicin	15	16.7
4	Doxorubicin + Cyclophosphamide+Docetaxel	16	17.8
5	Docetaxel + other antineoplastic agents	15	16.7
6	Other combinations of antineoplastic agents	20	22.2

**Table 4:** Relationships between CT (+, −) groups, subjective neuropsychological assessment variables, and therapeutic management.

		Variables of Subjective Neuropsychological Assessment					
		CT+	CT−	CT Cycles		Pharmacological Group 4	
				<4	≥4	Yes	No
**PCI**	Mean	50.60 ± 15.64	55.01 ± 12.10	52.92 ± 13.34	46.77 ± 14.84	44.96 ± 7.19	54.79 ± 12.11
	Median (IQR)	55 (22)	58 (20)	58.95 (25)	47 (23)	47(10)	60 (11)
	*p*-value	*p* = 0.005 ^a^		*p* = 0.002 ^a^		*p* = 0.013 ^a^	
**Oth**	Mean	14.18 ± 2.56	15.66 ± 1.44	15.13 ± 1.77	13.22 ± 2.88	14.81 ± 2.78	15.34 ± 1.45
	Median (IQR)	15 (3)	16 (0)	16 (1)	13 (4)	16 (1)	16 (0)
	*p*-value	*p* = 0.026 ^a^		*p* < 0.001 ^a^		*p* = 0.071	
**PCA**	Mean	13.30 ± 4.51	15.82 ± 4.22	15.01 ± 4.71	11.90 ± 3.81	13.67 ± 15.17	15.17 ± 4.37
	Median (IQR)	13.22 (5)	15 (7)	14 (6)	12 (6)	12.80 (8)	16.33 (5)
	*p*-value	*p* = 0.039 ^a^		*p* = 0.001 ^a^		*p* = 0.102	
**QoL**	Mean	11.73 ± 5.11	13.28 ± 4.76	10.93 ± 5.06	8.82 ± 3.56	10.19 ± 5.35	12.84 ± 4.12
	Median (IQR)	11 (6)	16 (4)	12.50 (10)	10 (3)	10.50 (9)	15 (6)
	*p*-value	*p* = 0.034 ^a^		*p* = 0.023 ^a^		*p* = 0.019 ^a^	
**PCI (<54)**	%	44.4%	28.9%	35%	52%	49.8%	56.3%
	χ^2^	χ^2^ = 19.29		χ^2^ = 7.018		χ^2^ = 1.44	
	*p*-value	*p* < 0.001 ^b^		*p* = 0.030 ^b^		*p* = 0.129	

Abbreviations: IQR, interquartile range; ^a^ *t*-test (178 degrees of freedom); ^b^ chi-square test (1 degree of freedom).

**Table 5:** Relationships between CT (+, −) groups and objective neuropsychological assessment variables.

Variables of Objective Neuropsychological Assessment									
		CT+	CT−	Number of CT Cycles	CT Cycles		PVF	SVF	rSO_2_
					<4	≥4			
**PVF**	Mean	41.26 ± 9.55	47.03 ± 9.31	-	45.00 ± 9.32	36.91 ± 8.20	na	-	-
	Median (IQR)	40 (14)	47.50 (14.75)	-	46 (14.5)	36 (11)	na	-	-
	*r* and/or *p*-value	*p* < 0.001 ^a^		*r* = −0.332 *p* < 0.001 ^b^	*p* < 0.001 ^a^		na	*p* = 0.592	*r* = −0.535 *p* < 0.001 ^b^
**SVF**	Mean	33.60 ± 10.44	36.14 ± 10.68		35.31 ± 10.68	31.47 ± 10.57	-	na	
	Median (IQR)	34 (17)	37.50 (17)		35 (17)	32 (17)	-	na	
	*r* and/or *p*-value	*p* < 0.001 ^a^		*r* = −0.154 *p* = 0.040 ^b^	*p* = 0.004 ^a^		*p* = 0.592	na	*r* = 0.485 *p* < 0.001 ^b^
**rSO_2_**	Mean	63.30 ± 8.02	67.98 ± 7.80	-	66.77 ± 7.47	62.88 ± 7.60	-	-	na
	Median (IQR)	62.58 (12.25)	68.66 (10.96)	-	68.41 (11.5)	62.66 (8.79)	-	-	na
	*r* and/or *p*-value	*p* < 0.001 ^a^		*r* = −0.225 *p* = 0.002 ^b^	*p* < 0.001 ^a^		*r* = 0.535 *p* < 0.001 ^b^	*r* = 0.485 *p* < 0.001 ^b^	na

Abbreviations: IQR, interquartile range; ^a^ *t*-test (178 degrees of freedom); ^b^ correlation test based on Pearson’s *r* coefficient (178 degrees of freedom).

**Table 6:** Evolution of rSO_2_ measures.

Measures (N = 66)	Left Side		Right Side		Both Sides	
	M ± SD	Correlation ^a^	M ± SD	Correlation ^a^	M ± SD	Correlation ^b^
rSO_2_-CT−_1_	64.81 ± 7.01	-	66.70 ± 8.01	-	65.75 ± 7.86	*r* = 0.841, *p* < 0.001
rSO_2_-CT−_2_	67.84 ± 7.22	*r* = 0.958, *p* < 0.001	69.89 ± 8.15	*r* = 0.805, *p* < 0.001	68.86 ± 8.52	*r* = 0.825, *p* < 0.001
rSO_2_-CT−_3_	67.13 ± 7.46	*r* = 0.907, *p* < 0.001	69.98 ± 9.11	*r* = 0.781, *p* < 0.001	68.55 ± 7.41	*r* = 0.795, *p* < 0.001
rSO_2_-CT−_4_	68.73 ± 7.62	*r* = 0.906, *p* < 0.001	68.85 ± 8.90	*r* = 0.730, *p* < 0.001	68.79 ± 7.02	*r* = 0.819, *p* < 0.001
rSO_2_-CT+_1_	60.69 ± 8.52	*r* = 0.616, *p* < 0.001	62.23 ± 9.01	*r* = 0.723, *p* < 0.001	61.46 ± 7.35	*r* = 0.743, *p* < 0.001
rSO_2_-CT+_2_	64.24 ± 7.29	*r* = 0.909, *p* < 0.001	65.19 ± 9.23	*r* = 0.829, *p* < 0.001	64.71 ± 8.36	*r* = 0.505, *p* < 0.001
rSO_2_-CT+_3_	63.14 ± 8.01	*r* = 0.909, *p* < 0.001	64.61 ± 9.01	*r* = 0.825, *p* < 0.001	63.87 ± 8.12	*r* = 0.521, *p* < 0.001
rSO_2_-CT+_4_	62.80 ± 8.23	*r* = 0.820, *p* < 0.001	63.53 ± 9.56	*r* = 0.912, *p* < 0.001	63.16 ± 8.62	*r* = 0.518, *p* < 0.001
M rSO_2_-CT−	67.12 ± 7.10	-	68.85 ± 8.32	-	67.98 ± 7.80	*r* = 0.821, *p* < 0.001
M rSO_2_-CT+	62.71 ± 8.01	*r* = 0.720, *p* < 0.001	63.89 ± 9.2	*r* = 0.628, *p* < 0.001	63.30 ± 8.02	*r* = 0.530, *p* < 0.001

Abbreviations: M, mean; SD, standard deviation. ^a^ *p*-Value corresponds to correlation with previous measure in the same side according to Pearson’s correlation test. ^b^ *p*-Value corresponds to correlation between both sides according to Pearson’s correlation test.

**Table 7:** Relationship between subjective and objective neuropsychological assessment variables.

Relation between Subjective and Objective Neuropsychological Assessment Variables			
	PVF	SVF	rSO_2_
**PCI**	*r* = 0.516 *p* < 0.001 ^a^	*r* = 0.630 *p* < 0.001 ^a^	*r* = 0.650 *p* < 0.001 ^a^
**Oth**	*r* = 0.170 *p* = 0.023 ^a^	*r* = 0.159 *p* = 0.033 ^a^	*r* = 0702 *p* < 0.001 ^a^
**PCA**	*r* = 0.675 *p* < 0.001 ^a^	*r* = 0.648 *p* < 0.001 ^a^	*r* = 0.395 *p* < 0.001 ^a^
**QoL**	*r* = 0.452 *p* < 0.001 ^a^	*r* = 0.153 *p* = 0.041 ^a^	*r* = 0.405 *p* < 0.001 ^a^
**PCI (<54)**	*p* < 0.001 ^b^	*p* < 0.001 ^b^	*p* < 0.001 ^b^

Abbreviations: ^a^ Correlation test based on Pearson’s r coefficient; ^b^ *t*-test.

## Data Availability

The data underlying this article cannot be shared publicly to maintain the privacy of individuals that participated in the study. The data will be shared on reasonable request to the corresponding author.
